# Crouzon’s Syndrome: A Rare Genetic Disorder

**DOI:** 10.5005/jp-journals-10005-1395

**Published:** 2016-12-05

**Authors:** Anupriya Kaushik, Hindpal Bhatia, Naresh Sharma

**Affiliations:** 1 Senior Lecturer, Department of Pedodontics and Preventive Dentistry, MN DAV Dental College, Solan, Himachal Pradesh, India; 2 Professor and Head, Department of Pedodontics and Preventive Dentistry, Manav Rachna Dental College, Faridabad, Haryana, India; 3 Reader, Department of Pedodontics and Preventive Dentistry, Manav Rachna Dental College, Faridabad, Haryana, India

**Keywords:** Crouzon’s syndrome, Fibroblast growth factor, Premature synostosis.

## Abstract

Crouzon’s syndrome, also known as brachial arch syndrome, is an autosomal dominant disorder with complete penetrance and variable expressivity. Described by a French neurosurgeon in 1912, it is a rare genetic disorder. Crouzon’s syndrome is caused by mutation in the fibroblast growth factor receptor 2 (FGFR2) gene. Normally, the sutures in the human skull fuse after the complete growth of the brain, but if any of these sutures close early, then it may interfere with the growth of the brain. The disease is characterized by craniosynostosis, with associated dentofacial anomalies. This report describes the different clinical features in a 10-year-old male patient, with particular reference to characteristic findings of this syndrome.

**How to cite this article:**

Kaushik A, Bhatia H, Sharma N. Crouzon’s Syndrome: A Rare Genetic Disorder. Int J Clin Pediatr Dent 2016;9(4):384-387.

## INTRODUCTION

Cranial skeletogenesis is a unique cranial malformation, although uncommon, compromising not only the function but also the mental well-being of the person.^1^ Described by a French neurosurgeon, Octave Crouzon, in 1912,^2^ it is a rare genetic disorder. It may be transmitted as an autosomal dominant genetic condition. Crouzon’s syndrome is caused by mutation in the fibroblast growth factor receptor 2 (FGFR2) genes.^3^

The disease is characterized by premature synostosis of coronal and sagittal sutures, which begins in the first year of life.^4^ Features of the condition include craniosynostosis, midfacial hypoplasia, maxillary retrusion, hypertelorism, and shallow orbits, which give rise to ocular proptosis.^5^ Intraoral manifestations include man-dibular prognathism, overcrowding of upper and lower teeth, and V-shaped maxillary dental arch.^2^ Narrow, high, or cleft palate and bifid uvula can also be seen. Occasional oligodontia, macrodontia, peg-shaped, and widely spaced teeth have been reported.^4^

Crouzon’s syndrome occurs in approximately 1 in 25,000 births worldwide.^6^ Crouzon’s syndrome makes up approximately 4.8% of all cases of craniosynostoses.^7^ No known race or sex predilection exists.^4^ The differential diagnosis of Crouzon’s syndrome includes simple craniosynostosis as well as Apert syndrome, Carpenter syndrome, Saethre-Chotzen syndrome, and Pfeiffer syndrome.^8^ The various abnormalities associated with Crouzon’s syndrome have been listed in Table 1.^9^

**Table 1:** **Table 1:** Abnormalities associated with Crouzon’s syndrome

*Cranium*	
Craniosynostosis	
Brachycephaly and acrocephaly	
Palpable ridge	
Flat occiput	
Frontal bossing	
*Facial features*	
Maxillary retrusion	
Malar deficiency	
Relative mandibular prognathism	
*Ear*	
Low set ear	
Conductive hearing loss	
Bilateral atresia of auditory meatus	
*Eye*	
Downslanting palpebral fissure	
Exophthalmos	
Iris - coloboma	
Ptosis	
Exposure keratitis	
Hypertelorism	
Divergent strabismus	
Nystagmus	
*Nose*	
Beaked nose	
Deviated nasal septum	
*Mouth*	
Short upper lip	
Class III malocclusion with maxillary crowding	
High arched and narrow palate	
Cleft palate and bifid uvula	
*Neurological*	
Headache	
Mild to moderate mental retardation	
Seizures	
*Musculoskeletal*	
Cervical spine abnormalities (scoliosis)	
Calcification of stylohyoid ligament	
Meniere’s disease (vertigo, dizziness, and/or ringing in the ear)	
*Respiratory system*	
Breathing difficulty	
Sleep apnea	
*Cutaneous*	
Acanthosis nigricans	

## CASE REPORT

A 10-year-old male patient, born by a full-term normal delivery, reported to our department along with his father. The chief complaint as reported by the father was pain in relation to the upper left tooth region of the child for the past 1 week. It was the patient’s first dental visit. Since the child’s appearance and head size were not normal, the family and medical history were taken in detail. No history of any systemic illness or drug allergy was reported by the patient’s father. There were no reported anomalies in any siblings or near relatives.

## EXTRAORAL EXAMINATION

**Fig. 1:**
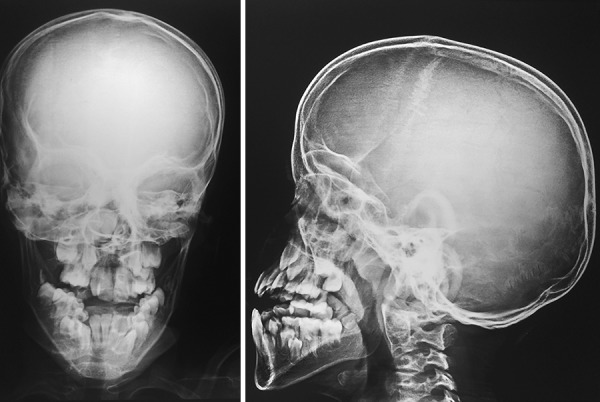
PA Skull view and Lateral skull view showing hypoplastic maxilla, relative mandibular prognathism and cerebral impressions

**Fig. 2:**
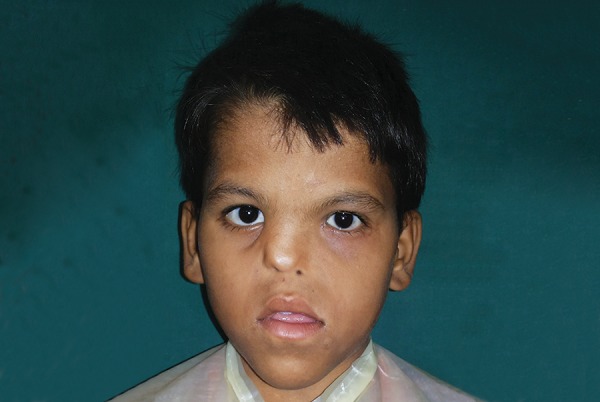
Front profile photograph showing depressed nasal bridge and deviated nasal septum

Clinical examination revealed hypertelorism, external strabismus, optical atrophy, hypoplastic malar process, hypoplastic maxilla, relative mandibular prognathism, depressed nasal bridge, deviated nasal septum, short upper lip, and long philtrum (Fig. 1). No digital abnormalities were present. Extraoral examination revealed elliptical-shaped head, with dolichofacial growth pattern, and concave facial profile (Fig. 2).

**Fig. 3:**
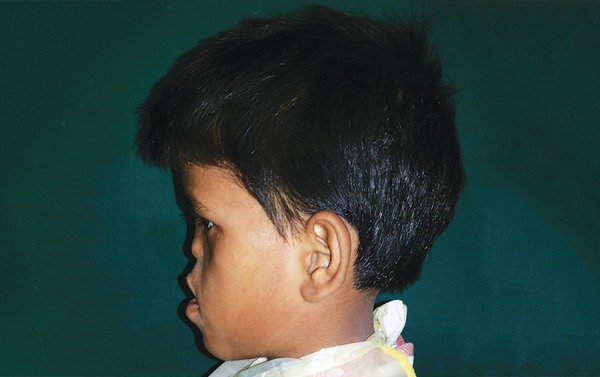
Right lateral profile photograph showing concave profile and relative mandibular prognathism

**Fig. 4:**
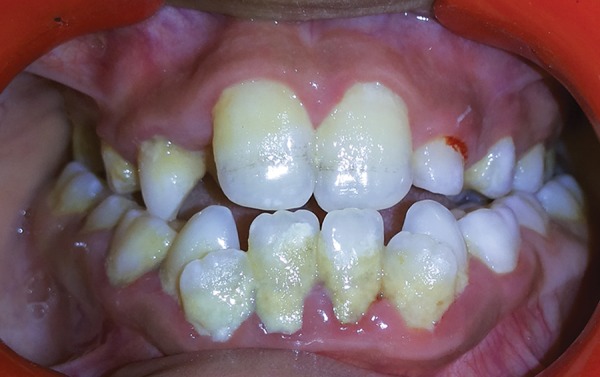
Intraoral photograph showing edge to edge incisal relationship

## INTRAORAL EXAMINATION

**Fig. 5:**
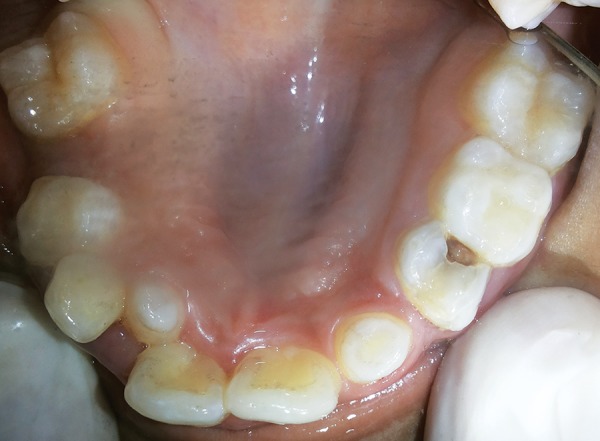
Intraoral photograph showing high arched palate, peg shaped laterals, missing 55 and carious 64

**Fig. 6:**
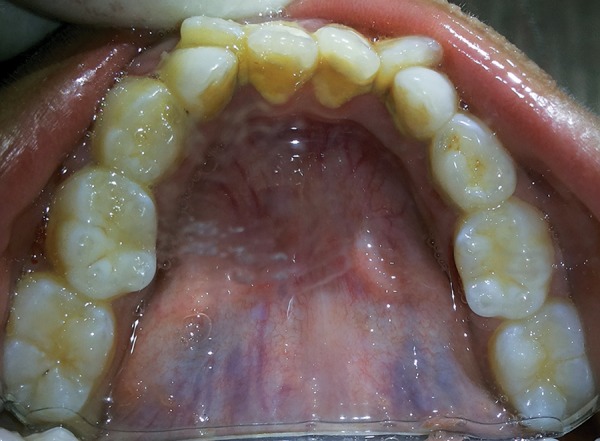
Intraoral photograph showing crowding wrt mandibular arch and grade III calculus in the labial segment

Intraoral examination revealed that the patient was in the late mixed dentition phase. There was apparent class III malocclusion with edge-to-edge incisal relationship with posterior crossbite on the right side and edge to edge on the left side (Fig. 3). Patient had V-shaped maxillary arch (Fig. 4), with crowding present especially in the lower labial segment (Fig. 5). The child also had high arched palate and bifid uvula, which are the characteristics of this syndrome (Fig. 6). Upper right deciduous second molar (55) and upper left deciduous canine (63) were missing. The tooth in question, i.e., upper left deciduous first molar (64), was found to be carious and grade II mobile (Fig. 4). Oral hygiene status was poor. Grade III calculus was evident, especially in the lower labial segment (Fig. 5).

## TREATMENT PLAN

**Fig. 7:**
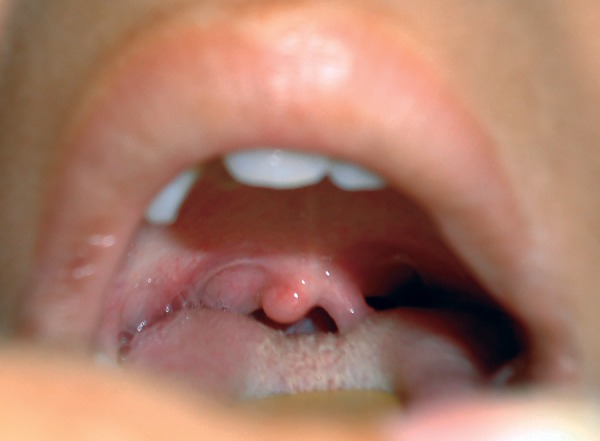
Intraoral photograph showing bifid uvula

**Fig. 8:**
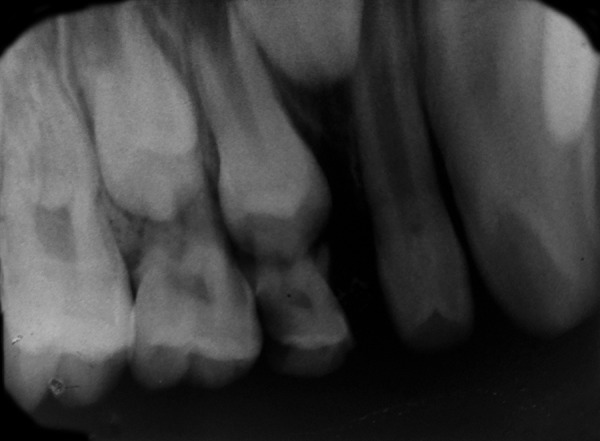
Intraoral periapical radiograph wrt 64 showing physiologic resorption

**Fig. 9:**
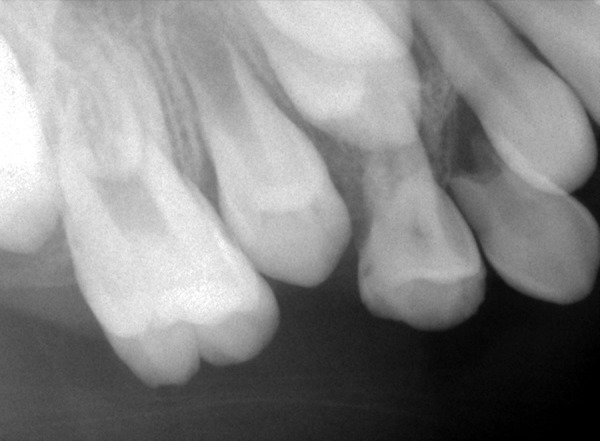
Radio visio graph wrt 55 region showing no overlying bone

**Fig. 10:**
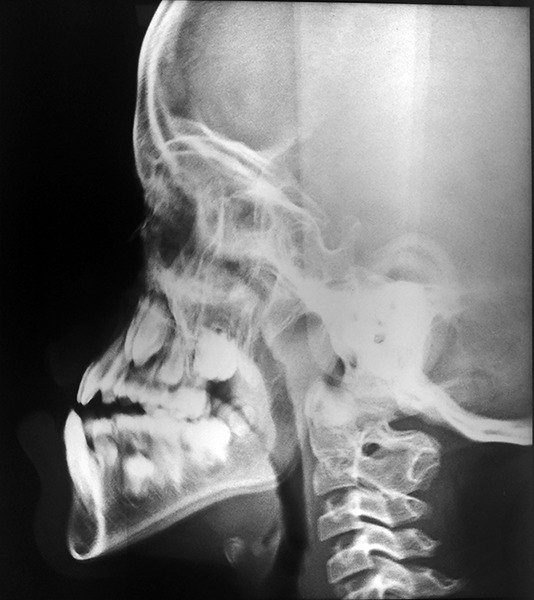
Lateral cephalogram of the patient showing fusion of cervical vertebra 2 and 3 (C2 and C3)

**Fig. 11:**
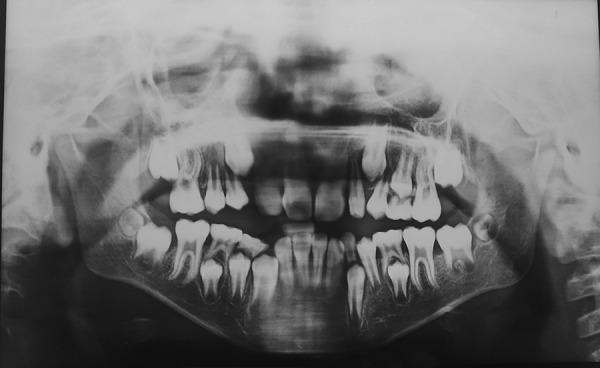
Postoperative intraoral photograph

**Fig. 12:**
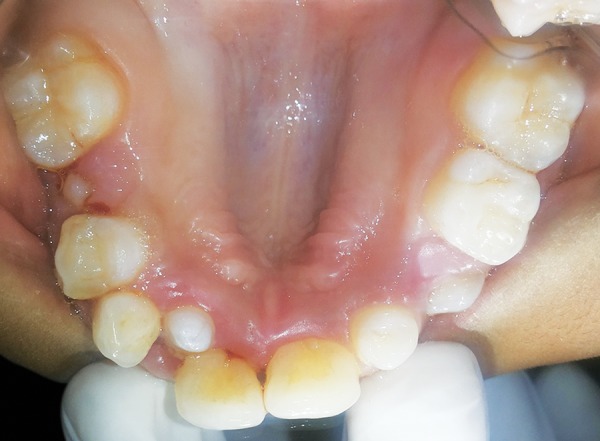
Follow-up photograph

First, intraoral periapical radiograph with respect to 64, lateral cephalogram, and lateral skull and posteroanterior skull views of the patient were advised; 64 region showed physiologic resorption of the concerned tooth (Fig. 7), and extraction of 64 was advised. Radiovisiography of 55 tooth region (Fig. 8) did not show bone overlying the erupting premolar, hence there was no need for the space maintainer in that region. Radiographic findings revealed hypoplastic maxilla, relative mandibular prog-nathism (Fig. 9), cerebriform impressions, hypophyseal fossa enlargement, and cervical region abnormalities, i.e., fusion of posterior bodies and elements (C2-C3) (Fig. 10). After clinical and radiographic investigations, complete oral prophylaxis was done, and the patient was advised to rinse twice daily with chlorhexidine mouthwash. Then extraction of 64 was performed under local anesthesia, and postextraction instructions were given with particular emphasis on lip biting, which is very common among children after being anesthetized. Also, ear, nose, and throat and ophthalmology opinion for the patient was taken. Patient was diagnosed to have congenital dacryocystitis along with slight visual impairment. Figures 11 and 12 show follow-up photographs. Digital panoramic radiograph of the patient was done at 6 months recall, which revealed normal eruption of the succedaneous teeth.

## DISCUSSION

The phenotypic features of Crouzon’s syndrome may be absent at birth and may evolve gradually during the first few years of life.^10,11^ It is commonly inherited as an autosomal dominant trait, with complete penetrance and a variable expressivity, but about one third of the cases do arise spontaneously. The male to female preponderance is 3:1.^12^ With the advent of molecular technologies, the gene for Crouzon’s syndrome could be localized to the FGFR2 gene, at the chromosomal locus 10q25.3-q26, and more than 30 different mutations within the gene have been documented in separate families.^13^ Hypertelorism was a universal finding in the affected individuals and is thought to arise due to a decrease in growth of the sphenozygomatic and sphenotemporal sutures.^5^ The appearance of an infant with Crouzon’s syndrome can vary in severity from a mild presentation with subtle midface deficiency to severe forms with multiple cranial sutures fused and marked midface and eye problems. Upper airway obstruction can lead to acute respiratory distress and the presence of mental retardation is rare in these children.^14^

## CONCLUSION

An understanding of these abnormalities is necessary for the dental team to make the appropriate referrals to ensure that the patient receives the best available care. The pediatric dentist should be an integral part of the multidisciplinary team.
